# The Relationship between Sparseness and Energy Consumption of Neural Networks

**DOI:** 10.1155/2020/8848901

**Published:** 2020-11-25

**Authors:** Guanzheng Wang, Rubin Wang, Wanzeng Kong, Jianhai Zhang

**Affiliations:** ^1^Institute for Cognitive Neurodynamics, School of Science, East China University of Science and Technology, Meilong Road 130 Shanghai 200237, China; ^2^Key Laboratory of Brain Machine Collaborative Intelligence of Zhejiang Province, Hangzhou Dianzi University, Zhejiang, China

## Abstract

About 50-80% of total energy is consumed by signaling in neural networks. A neural network consumes much energy if there are many active neurons in the network. If there are few active neurons in a neural network, the network consumes very little energy. The ratio of active neurons to all neurons of a neural network, that is, the sparseness, affects the energy consumption of a neural network. Laughlin's studies show that the sparseness of an energy-efficient code depends on the balance between signaling and fixed costs. Laughlin did not give an exact ratio of signaling to fixed costs, nor did they give the ratio of active neurons to all neurons in most energy-efficient neural networks. In this paper, we calculated the ratio of signaling costs to fixed costs by the data from physiology experiments. The ratio of signaling costs to fixed costs is between 1.3 and 2.1. We calculated the ratio of active neurons to all neurons in most energy-efficient neural networks. The ratio of active neurons to all neurons in neural networks is between 0.3 and 0.4. Our results are consistent with the data from many relevant physiological experiments, indicating that the model used in this paper may meet neural coding under real conditions. The calculation results of this paper may be helpful to the study of neural coding.

## 1. Introduction

Recent studies have shown that single neuron firing is sufficient to influence learning and behavior [1, 2]. The result challenges people's long-standing understanding that a behavioral response needs the firing of thousands of neurons. Their findings provide the basis and support for a neural theory (neuron “sparse coding” hypothesis); the hypothesis argues that a small number of neurons are enough to encode information [3–5]. Only a small part of neurons are activated when signaling in a sparse coding mode, and most of the neurons are responsible only for network connection [6–8]. Since a small number of neuron firing and little energy are required in the sparse coding mode, the sparse coding is an energy-efficient neural coding method [9, 10]. This energy-efficient neural coding pattern increases the ratio of neuron-encoded information and greatly improves energy efficiency [11, 12]. Although the sparse coding hypothesis of neural networks in the cerebral cortex has not yet been confirmed, it has been shown that sparse coding represents the maximization of energy efficiency [13–15].

Wang et al. studied the information carried by neurons and the energy cost by neurons [13]. They found that neurons are not most energy-efficient when coding the maximum information, and the ratio of signaling to fixed costs affects the total energy consumed by neurons. Laughlin studied the sparseness and representational capabilities of neural networks [14]. They found that the sparseness of the most energy-efficient coding pattern depends on the ratio of signaling to fixed costs when neural networks have similar representational capabilities. However, Wang et al. and Laughlin did not consider the exact ratio of signaling to fixed costs. Wang et al.'s study believes that the ratio of signaling to fixed costs is between 10 and 200, and Laughlin just studied three cases with a ratio of 1, 10, and 100. They just considered a very large range of ratios and did not give the sparseness of the most energy-efficient coding mode. The purpose of this paper is to find the sparseness of the most energy-efficient coding mode based on the research of Wang et al.

In this paper, we first study the relationship among the total energy cost of neural networks, the ratio of signaling to fixed cost, and the ratio of active neurons to all neurons in networks under similar representational capabilities. We found that the ratio of active neurons to all neurons in most energy-efficient neural networks is related to the ratio of signaling to fixed costs. When the ratio of signaling to fixed costs is high (70~100), the optimal ratio of active neurons to all neurons in neural networks (at this time, neural networks cost the least energy) is less than 0.1. When the ratio of signaling to fixed costs is low (1~20), the optimal ratio of active neurons to all neurons in neural networks is between 0.2 and 0.5.

Based on the above work, we calculated the ratio of signaling to fixed costs by the data from physiological experiments and gave an exact ratio signaling to fixed costs. We studied the relationship between the total energy cost of different neural networks and the ratio of active neurons to all neurons when neural networks have similar representational capabilities. We found that the total energy cost of a network is the least when the ratio of active neurons to all neurons is between 0.3 and 0.4. In addition, neural networks have the most representational capabilities with the same total energy cost when the ratio of active neurons to all neurons is between 0.3 and 0.4. This paper confirms that neural networks have the most representational capabilities and the least total energy cost when the ratio of active neurons to all neurons is between 0.3 and 0.4.

Compared with the published research, we give an exact ratio of signaling to fixed costs and calculate the ratio of signaling costs to total energy costs of neural networks. We give the optimal ratio of active neurons to all neurons in neural networks with the most representational capabilities and the least total energy cost. The significance of this paper is not only to determine the ratio of signaling to fixed costs but also to prove that the sparse coding mode is a kind of energy-saving neural coding mode. This mode is in line with the maximization of neural signal transmission theory and the maximization of energy utilization rate theory [16–18]. In addition, the ratio of signaling costs to total energy costs of neural networks and the ratio of active neurons to all neurons in neural networks are consistent with the results of the correlation physiology experiments [19, 20]. This suggests that the model of neural networks that we used is likely to conform to the coding of neurons under real conditions, and the results may be helpful in the study of neural coding theory.

## 2. Model

The representational capacity (cap) of neural networks refers to the number of active neurons arranged in a neural network, depending on the total number of neurons *N* and the number of active neurons *A*. The equation for calculating cap is given by

(1) $$\begin{array}{c} \text{cap}=\frac{N!}{A!\left(N-A\right)!}. \end{array}$$

The ratio *r* is the ratio of signaling to fixed costs; it depends on a single neuron signaling cost (ac) in unit time divided by fixed costs (fc). The ratio *r* is given by

(2) $$\begin{array}{c} r=\frac{\text{ac}}{\text{fc}}. \end{array}$$

The total energy consumption of neural networks (cost) is the sum of fixed energy costs of all neurons and signaling costs of active neurons, and the cost is given by

(3) $$\begin{array}{c} \text{cost}=\text{fc}\times N+\text{ac}\times A. \end{array}$$

The ratio of active neurons to all neurons in a neural network (*p*) is the number of active neurons divided by the number of neurons *N* in the network; *p* is given by

(4) $$\begin{array}{c} p=\frac{A}{N}. \end{array}$$

**Figure 1:**
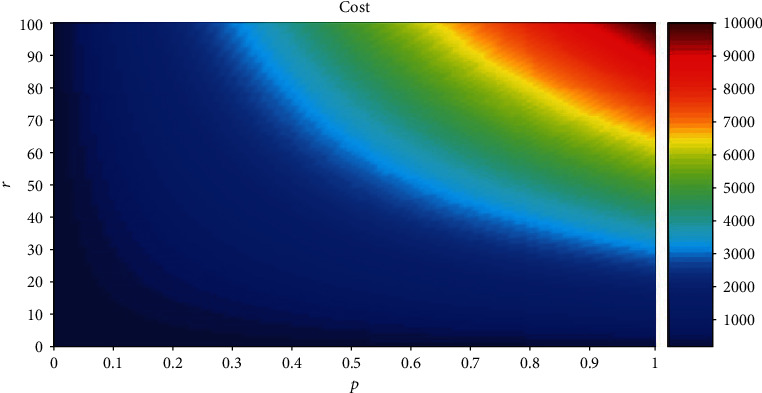
The relationship between cost and *p* and *r*.

From equations (2), (3), and (4), we can get the relationship between costs, *p* and *r*, given by

(5) $$\begin{array}{c} \text{cost}=\text{fc}\times N+r\times \text{fc}\times N\times p=\text{fc}\times N\times \left(1+r\times p\right), \end{array}$$

where fc is a fixed constant, and if we fixed the total number of neurons in a neural network, then fc and *N* do not affect the total energy consumption. If we disregard the representational capacity and just consider the total energy consumption, the ratio of active neurons to all neurons, and the ratio of signaling to fixed costs, then we can find that the total energy consumption and the ratio of active neurons to all neurons are linear-related and the total energy consumption and the ratio of signaling to fixed costs are linear-related. For simplicity, fc is set to 1 and *N* is set to 100. The ratio *p* is between 0 and 1, and related research believed that the ratio *r* is between 1 and 100 [13, 14]. When *p* is a fixed value, the cost increases linearly with the increase of *r*, as shown in Figure 1. Note that we do not consider the representational capacity.

The horizontal axis is *p*, the vertical axis is *r*, the color is cost, blue means the total energy consumption is lower, red means the total energy consumption is higher, *N* is 100, and fc is 1.

It can be seen that if we do not consider the representational capacity when fc and *N* are fixed, the cost is linearly increased as *p* and *r*. Now, we consider representational capacity. From equation (1), we can see that when cap is fixed, if *A* is determined, then *N* can be obtained according to the value of *A*. According to equation (4), we can obtain *p*. In other words, *N* and *p* are not independent variables when cap is a fixed value. From equation (5), although *N* is a variable value, cost is only related to *r* and *p* when cap is a fixed value.

**Figure 2:**
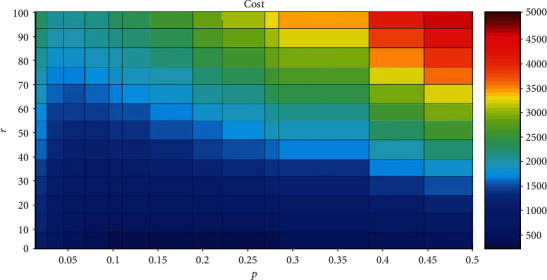
The relationship between cost and *p* and *r*.

Table 1 shows the number of total neurons and the corresponding number of active neurons in neural networks which have approximately representational capacity. When *N* is 100, *A* is 50, and *p* is 0.5, from equation (1), we can get that the corresponding cap is 1.0089*e* + 029. We use 1.0089*e* + 029 as a benchmark, and the normalization technique is adopted for cap. There are many cases which have a different number of neurons, different number of active neurons, and different ratio of active neurons to all neurons. The difference between these cases and the above benchmark value is within 5%. Although *N* is variable, cost is just related to *r* and *p*. The relationship between cost and *r* and *p* is shown in Figure 2.

These cases have different *N*, different *A*, and different *p* and have approximately representational capacity. We normalize the value of the first cap as a benchmark. The difference between these cases and the above benchmark value is within 5%. Since these cap values are difficult to exactly match, we consider these cases to have the same value of representational capacity.

The horizontal axis is *p*, and the range is 0 to 0.5. The vertical axis is *r*, and the range is 1~100. The horizontal axis has not taken into account the range of 0.5 to 1, because when *p* exceeds 0.5, we can find*p* below 0.5, and they have the same representational capacity. For example, *p* = 0.8 and *p* = 0.2 have the same representational capacity, but the total energy cost is high when *p* = 0.8, so the horizontal axis has not taken into account the range of 0.5 to 1.

As can be seen from Figure 2, if the value of *r* is large, the value of cost will be large when *p* is the same. When *p* is large (0.4~0.5), the cost is greatly affected by *r*. When *p* is small (0.05~0.1), the cost is hardly influenced by *r*. This is because when *p* is large, the number of active neurons in neural networks is large. When *r* is large, it means that the signaling cost is much larger than the fixed cost, so the total energy cost of the network will be large. When *p* is small, the number of active neurons in neural networks is small and the energy cost of signaling is small, so the total energy cost of the networks is hardly affected by *r*.

In Figure 2, when the value of *r* is different, the value of *p* is also different corresponding to the minimum value of cost. When *r* is large (70~100) and *p* is around 0.05, the value of cost is the least. When *r* is small (0~20) and *p* is between 0.1 and 0.4, the value of cost is the least. Therefore, the value of *p* corresponding to the minimum cost depends on the value of *r*. Considering the published results, the range of *r* in Figure 2 is set to 1 to 100, but this range is rough. We will give a more precise range of *r* by the data from physiological experiments.

From equation (2), we can see that the value of *r* depends on signaling cost and fixed cost. The cost of signaling includes the generation of a spike, propagation along the axon, mechanisms of transmitter release, and recycling [21]. Studies have shown that the generation of spikes and the propagation of action potentials account for about 95% of the total energy cost of signaling [21, 22]. In this study, we consider that the energy cost of signaling just includes the generation of spikes and the propagation of action potentials.

There are a lot of glial cells in the brain besides neurons; glial cells provide support and protection for neurons and supply nutrients and oxygen to neurons [22]. A glial cell has no axons, and it cannot generate a spike [22, 23]. Neuron activity requires glial cells to provide nutritional support, and the costs of glial cells need to be included in the fixed costs of neural networks. Therefore, the fixed costs of neural networks include the cost of maintaining resting potentials in neurons and glial cells. That is, the total energy consumption of neural networks includes the cost of maintaining resting potentials in neurons and glial cells, the generation of spikes, and the propagation of action potentials.

According to the relevant experimental results [24], the generation of one spike in one neuron needs to cost 3.84 × 10^8^ ATPs; 3.28 × 10^8^ ATPs support action potential propagation to output synapses along axon collaterals. About 3.42 × 10^8^ ATPs/s are used to maintain the resting potential of a neuron; 1.02 × 10^8^ ATPs/s are used to maintain the resting potential of a glial cell. For neuron populations in chick retinal ganglion cells, the mean action potential frequency range is between 3 and 4 Hz [20]. Therefore, we use the boundary value of the mean rate, 3~4 Hz. It is generally believed that the number of glial cells in the brain is 10 times more than the number of neurons, so in this study, the ratio of the number of neurons to glial cells is 1 : 10.

Based on the above data, when the mean action potential frequency in neural networks is 3 Hz (or 4 Hz), the signaling cost of a single neuron per second is (3.84 × 10^8^ + 3.28 × 10^8^) × 3 (or 4) = 21.36 × 10^8^ (28.48 × 10^8^) ATPs and the fixed cost of a single neuron and ten glial cells is (3.42 × 10^8^ + 10 × 1.02 × 10^8^) = 13.62 × 10^8^ ATPs per second. The total energy cost is 21.36 × 10^8^ (28.48 × 10^8^) + 13.62 × 10^8^ = 34.98 × 10^8^ (42.1 × 10^8^) ATPs per second. According to the above results, the cost of signaling accounted for 61% to 68% of the total energy cost, which matches Sokoloff experimental results (the cost of signaling accounted for 50% to 70% of total energy consumption) [25]. This shows that the above calculation is reasonable and credible.

Note that the neural networks mentioned herein just include neurons and do not include glial cells. Because the glial cells do not release the action potential, nor directly involve in the coding, the glial cells were ignored in the calculation of coding ability of neural networks. Glial cells were just included in the calculation of fixed cost.

**Figure 3:**
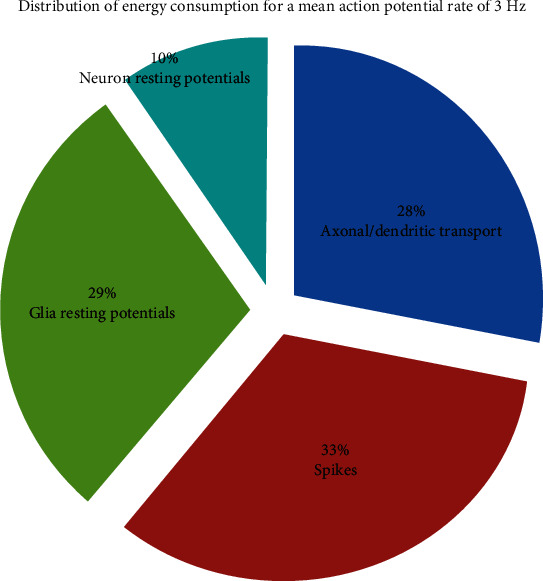
Distribution of energy consumption in neural networks.

**Figure 4:**
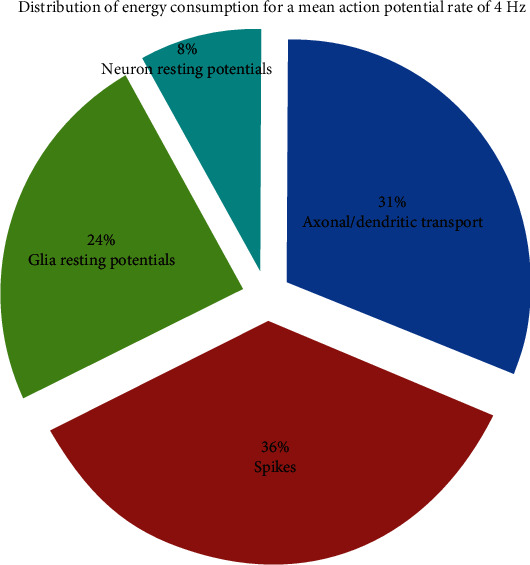
Distribution of energy consumption in neural networks.

When the mean action potential frequency in neural networks is 3 Hz or 4 Hz, the ratio of the cost of maintaining resting potentials in neurons and glial cells, the generation of spikes, and the propagation of action potentials to the total energy cost are shown in Figures 3 and 4, respectively.

When the mean action potential frequency in neural networks is 3 Hz, at the cost of maintaining resting potentials in neurons and glial cells, the generation of a spike and the propagation of action potentials account for 10%, 29%, 33%, and 28% of the total energy consumption, respectively.

When the mean action potential frequency in neural networks is 4 Hz, at the cost of maintaining resting potentials in neurons and glial cells, the generation of spikes and the propagation of action potentials account for 8%, 24%, 36%, and 31% of the total energy consumption, respectively.

Based on the above data, we can calculate *r* when the mean action potential frequency is 3 Hz or 4 Hz according to equation (2):

(6) $$\begin{array}{c} r=\frac{\text{ac}}{\text{fc}}=\frac{\left(3.84\times 10^{8}+3.28\times 10^{8}\right)\times 3\left(\text{or }4\right)}{3.42\times 10^{8}+10\times 1.02\times 10^{8}}=1.57\left(\text{or }2.09\right). \end{array}$$

**Figure 5:**
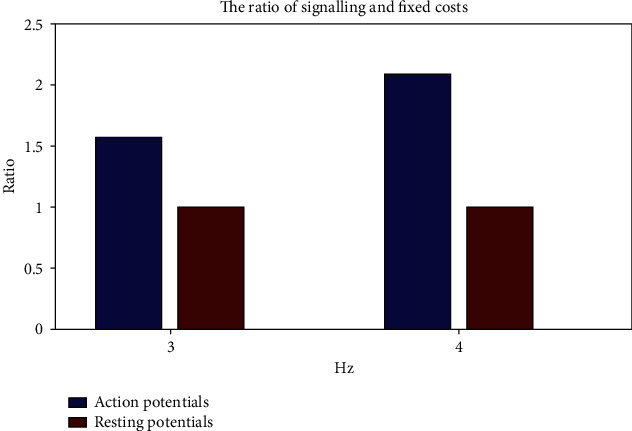
The ratio of signaling cost to fixed energy cost.

The ratio of signaling cost to fixed cost, that is, the values of *r*, is shown in Figure 5 when the mean action potential frequency is 3 Hz or 4 Hz.

The blue pillar is signaling cost, and the red pillar is fixed cost. The horizontal axis is the action potential frequency, and the vertical axis is the ratio of signaling cost to fixed cost, which is 1.57 and 2.09, respectively.

In a real situation, the number of glial cells is above 10 times more than the number of neurons. That is, the value of *r* is lower than that we calculated, so we set that the range of *r* is 1.3 to 2.1.

We set a fixed value of *r* in the range of 1.3 to 2.1, then calculate the representational capacity of different neural networks with different *N* and different *A* which have the same total energy consumption. To facilitate the calculation, fc is set to 1. The value of ac is equal to the value of *r* through equation (2). According to equation (3), we can get

(7) $$\begin{array}{c} \text{cost}=\text{fc}\times N+\text{ac}\times A=N+r\times A, \end{array}$$

where cost and *r* are constant; so, we can get

(8) $$\begin{array}{c} N=-r\times A+\text{cost}. \end{array}$$

**Figure 6:**
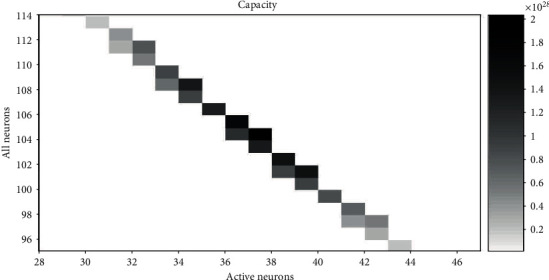
The representational capacity of different neural networks.

**Figure 7:**
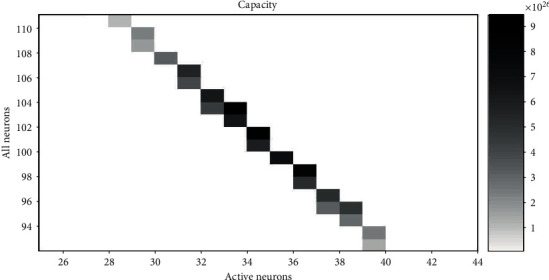
The representational capacity of different neural networks.

**Figure 8:**
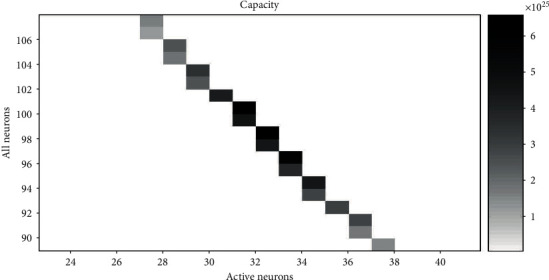
The representational capacity of different neural networks.

**Figure 9:**
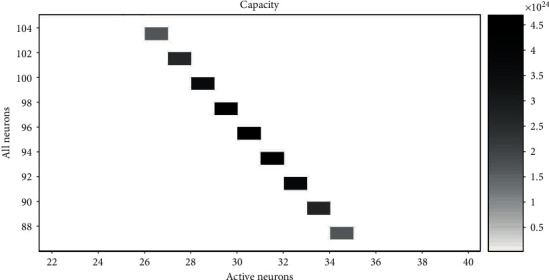
The representational capacity of different neural networks.

The corresponding representational capacity and the ratio of active neurons to all neurons can be calculated according to equations (1) and (2). Therefore, we can get the value *N* and the value *A* with the maximum cap when cost is fixed. When the range of the cost is 154 to 156 (within a difference of 2%, approximately equal) and the value of *r* is 1.4, 1.6, 1.8, and 2.0, the relationship between *N* and *A* and cap is shown in Figures 6–9, respectively. The squares in Figures 6–9 show the neural networks with different total neurons (*N*) and different numbers of active neurons (*A*). The cost of these networks is between 154 and 156, and the color depth indicates the value of the corresponding representational capacity. The color is deep which means cap is large. The value of *N* corresponding to the maximum cap in Figure 6 is 104, the value of *A* is 37, and the value of *p* is 37/104 = 35.6%. The value of *N* corresponding to the maximum cap in Figure 7 is 103, the value of *A* is 33, and the value of *p* is 33/103 = 32.0%. The value of *N* corresponding to the maximum cap in Figure 8 is 100, the value of *A* is 31, and the value of *p* is 31/100 = 31.0%. The value of *N* corresponding to the maximum cap in Figure 9 is 95, the value of *A* is 30, and the value of *p* is 30/95 = 31.6%.

It can be seen that when *r* is different, cap of the network is different though the value of cost is the same. When *r* is 1.4, 1.6, 1.8, and 2.0, the value of *p* of the network corresponding to the maximum cap is 35.6%, 32%, 31%, and 31.6%, respectively. However, since both *N* and *A* must be positive integers in equation (8), the value of cost affects the values of *N* and *A*, which will cause the value of *p* to be discontinuous. Therefore, the value of *p* we get may not be accurate.

A small rectangular block in the figure represents neural networks with total energy cost between 154 and 156. The horizontal axis is the number of active neurons in the network; the vertical axis is the total number of neurons in the network. The color of the small rectangle indicates the representational capacity of the network. Black means large representational capacity, and white color means small representational capacity. The case that corresponds to the maximum representational capacity is *N* = 104, *A* = 37, and *p* = 37/104 = 35.6%.

A small rectangular block in the figure represents neural networks with total energy cost between 154 and 156. The horizontal axis is the number of active neurons in the network; the vertical axis is the total number of neurons in the network. The color of the small rectangle indicates the representational capacity of the network. Black means large representational capacity, and white color means small representational capacity. The case that corresponds to the maximum representational capacity is *N* = 103, *A* = 33, and *p* = 33/103 = 32.0%.

A small rectangular block in the figure represents neural networks with total energy cost between 154 and 156. The horizontal axis is the number of active neurons in the network; the vertical axis is the total number of neurons in the network. The color of the small rectangle indicates the representational capacity of the network. Black means large representational capacity, and white color means small representational capacity. The case that corresponds to the maximum representational capacity is *N* = 100, *A* = 31, and *p* = 31/100 = 31.0%.

A small rectangular block in the figure represents neural networks with total energy cost between 154 and 156. The horizontal axis is the number of active neurons in the network; the vertical axis is the total number of neurons in the network. The color of the small rectangle indicates the representational capacity of the network. Black means large representational capacity, and white color means small representational capacity. The case that corresponds to the maximum representational capacity is *N* = 95, *A* = 30, and *p* = 30/95 = 31.6%.

The values of *p* that are calculated in Figures 6–9 are not continuous and are related to the total number of neurons in neural networks. We need to let the representational capacity just related to *p* and regardless of the total number of neurons *N*. We take the logarithm of cap which does not affect the comparison of representational capacity of different networks by the Stirling equation (see equation (9)). In this case, the ratio of cap to the cost is not related to *N* in neural networks [26]:

(9) $$\begin{array}{c} n!\approx \sqrt{2\pi n}\times {\left(\frac{n}{e}\right)}^{n}. \end{array}$$

**Figure 10:**
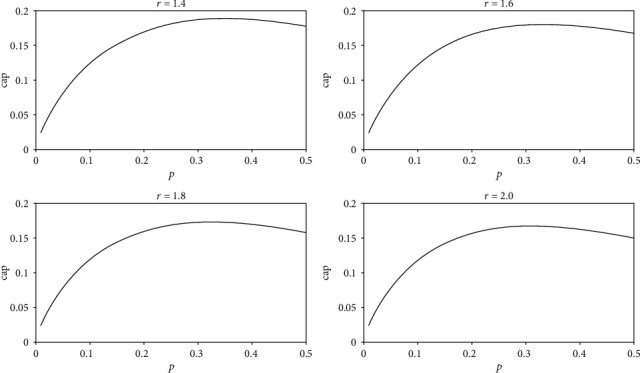
The relationship between cap and *p*.

**Figure 11:**
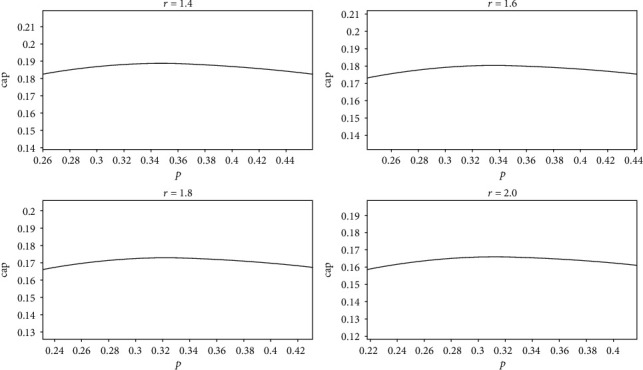
The relationship between cap and *p*.

From representational capacity cap = ln[*N*!/(*Np*)!×(*N* − *Np*)!], we can obtain the ratio of cap to the cost which is not related to *N* by equation (9):

(10) $$\begin{array}{c} \text{cap}=N\times \left[-p\times \ln p-\left(1-p\right)\times \ln\left(1-p\right)\right]-0.5\times \ln N-C, \end{array}$$

where *C* = 0.5 × ln[2*p* × (1 − *p*)] is a constant. When *N* is large, 0.5 × ln*N*/*N* also tends to 0. These two parts are not affecting the calculation of cap and can be ignored. The equation for calculating the ratio of cap to cost is given by

(11) $$\begin{array}{c} \frac{\text{cap}}{\text{cost}}=\frac{-p\times \ln p-\left(1-p\right)\times \ln\left(1-p\right)}{\text{fc}\times \left(1+r\times p\right)}, \end{array}$$

where the fixed cost fc is a constant. When cost and *r* are fixed, cap is only related to *p* and cap is not related to *N*. Let fc = 1 and cost = 1; the relationship between cap and *p* obtained by equation (11) is shown in Figure 10. All four graphs in Figure 10 show that cap increases first and then decreases with the increase of *p*. When *p* is between 0.3 and 0.4, cap is maximized. To facilitate observation, we enlarge a part of the graphs in Figure 10, as shown in Figure 11. It can be seen that the values of *p* corresponding to the maximum cap are different, but all the values of *p* are between 0.3 and 0.4. That is, when *r* is 1.4 or 1.6 or 1.8 or 2.0, a neural network has the largest representational capacity if the ratio of active neurons to all neurons is between 30% and 40%.

The horizontal axis is *p*, and the vertical axis is cap. The values of *r* in the four graphs are 1.4, 1.6, 1.8, and 2.0, respectively. In equation (11), both fc and cost are equal to 1. The relationship between cap and *p* is as follows:

(12) $$\begin{array}{c} \text{cap}=\frac{-p\times \ln p-\left(1-p\right)\times \ln\left(1-p\right)}{1+r\times p}. \end{array}$$

The portions where *p* is between 0.3 and 0.4 in Figure 10 are enlarged. The four pictures correspond to the four pictures in Figure 10, respectively. The maximum values of cap are 0.1887, 0.1803, 0.1728, and 0.1660, and the corresponding *p* are 0.353, 0.341, 0.329, and 0.319, respectively.

**Figure 12:**
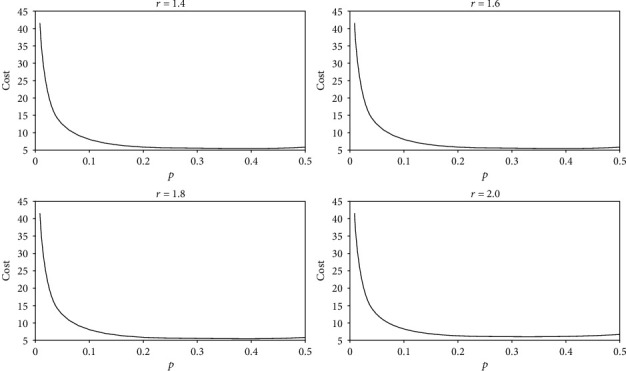
The relationship between cost and *p*.

**Figure 13:**
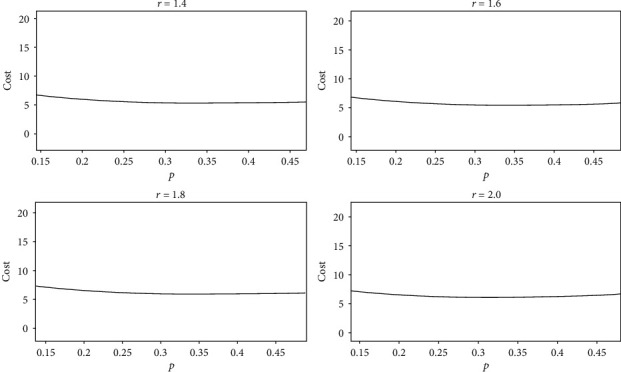
The relationship between cost and *p*.

According to equation (11), when cap and *r* are fixed, cost is only related to *p*; the relationship between cost and *p* is shown in Figure 12. Figures 12 and 10 are the opposite; with the increase of *p*, costs first decrease and then increase. The value of cost reaches the minimum when *p* is between 0.3 and 0.4. We enlarge part of the graphs in Figure 12, as shown in Figure 13. The value of *p* that corresponds to the minimum cost in Figure 13 is the same as the value of *p* that corresponds to the maximum cap in Figure 11. That is, when *r* is 1.4 or 1.6 or 1.8 or 2.0, a neural network has the smallest total cost if the ratio of active neurons to all neurons is between 30% and 40% and the sparse ratio of 30% to 40%.

The horizontal axis is *p*, and the vertical axis is cost. The values of *r* in the four graphs are 1.4, 1.6, 1.8, and 2.0, respectively. In equation (11), both fc and cap are equal to 1. The relationship between cost and *p* is as follows:

(13) $$\begin{array}{c} \cos\text{t}=\frac{1+r\times p}{-p\times \ln p-\left(1-p\right)\times \ln\left(1-p\right)}. \end{array}$$

The portions where *p* is between 0.3 and 0.4 in Figure 12 are enlarged. The four pictures correspond to the four pictures in Figure 12, respectively. The minimum values of cost are 5.2994, 5.5463, 5.7870, and 6.0241, and the corresponding *p* are 0.353, 0.341, 0.329, and 0.319, respectively.

## 3. Results

In Figure 10, we give four special values of *r* and get the value of *p* which corresponds to the maximum value of cap. All the values of *p* are between 0.3 and 0.4. It is unclear whether the values of *p* corresponding to the maximum cap are between 0.3 and 0.4 when *r* is between 1.3 and 2.1. In Figure 12, it is unclear whether the values of *p* corresponding to the minimum cost are between 0.3 and 0.4 when *r* is between 1.3 and 2.1. We need to find out the relationship among cost, cap, and *p* when *r* is a continuous change between 1.3 and 2.1.

**Figure 14:**
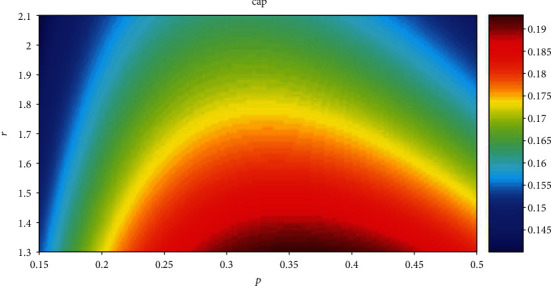
The relationship between cap and *p* and *r*.

**Figure 15:**
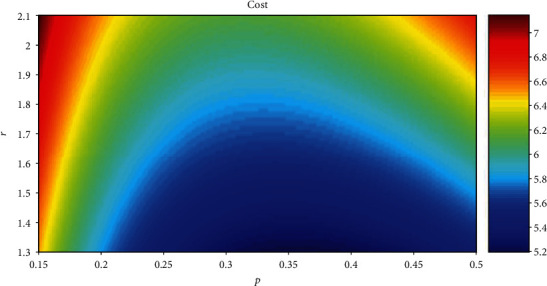
The relationship between cost and *p* and *r*.

Figure 14 shows the relationship among cap, *p*, and *r* when the cost is the same. For convenience, fc is set to 1, and cost is set to 1 in equation (11). The statistical results show that when *r* is the same, all the values of *p* corresponding to the maximum cap are between 0.3 and 0.4.  Figure 15 shows the relationship among cost, *p*, and *r* when cap is the same. The statistical results show that when *r* is the same, all the values of *p* corresponding to the minimum cost are between 0.3 and 0.4.

The horizontal axis is *p*, the vertical axis is *r*, and the color corresponds to the value of cap. The red area indicates high value of caps, and the blue area indicates low value of caps. For convenience, fc and cost are set to 1 in equation (11). The relationship between cap and *p* iscap = (−*p* × ln*p* − (1 − *p*) × ln(1 − *p*))/(1 + *r* × *p*).

The horizontal axis is *p*, the vertical axis is *r*, and the color corresponds to the value of cost. The red area indicates the high value of cost, and the blue area indicates the low value of cost. For convenience, fc and cap are set to 1 in equation (11). The relationship between cost and *p* is cost = (1 + *r* × *p*)/(−*p* × ln*p* − (1 − *p*) × ln(1 − *p*)).

Figure 14 shows that all the values of *p* corresponding to the maximum cap are between 0.3 and 0.4 when the cost is the same.  Figure 15 shows that all the values of *p* corresponding to the minimum cost are between 0.3 and 0.4 when cap is the same. It is uncertain whether the value of *p* which is between 0.3 and 0.4 corresponds to the minimum cost and maximum cap at the same time. According to equation (11), the change trend of cap/cost is the same as the change trend of cap in Figure 14. That is, the value of *p* which is between 0.3 and 0.4 corresponds to the minimum cost and maximum cap at the same time and has no relation with the total number of neurons.

## 4. Discussion

The generation of spikes and the propagation of action potentials consume much energy, in total accounting for about 50% to 70% of the total energy cost by neural networks [24–27]. The less the number of active neurons in a neural network, the less energy the network cost. Studies have shown that sparse neural coding patterns reflect the maximization of energy efficiency, that is, consume little energy to encode information [9, 28–30].

In this paper, we first calculate the ratio of signaling to fixed costs according to the data from physiological experiments, and the ratio is between 1.3 and 2.1. We find the generation of spikes and the propagation of action potentials accounted for about 56% to 68% of total energy consumption, and the results are consistent with existing experimental results. It confirms that the calculated results are believable and meet the energy consumption of neural networks under physiological experiments [24, 25].

Secondly, we simulate the relationship between the ratio of active neurons to all neurons in neural networks and the total energy consumption of networks with the same representational capacity. Statistical analysis shows that neural networks have the least total energy consumption if the ratio of active neurons to all neurons is between 30% and 40%. We simulate the relationship between the ratio of active neurons to all neurons in neural networks and the total energy consumption of networks with the same representational capacity. Statistical analysis shows that neural networks have the largest representational capacity if the ratio of active neurons to all neurons is between 30% and 40%. These two simulations are more consistent with physiological experiments (Zhang and Rochefort's study on the chick retina and mouse visual cortex, respectively. They found that the ratios of active neurons to all neurons are 33% and 36%, respectively [18–20]). We derive the optimal sparse proportion of active neurons in neuron clusters through real physiological data and rigorous formulas. The physiological significance of this result is that the proportion of active neurons can be determined in subsequent studies or in designing neuron cluster experiments. In addition, the ratio of the energy consumed by the neurons to the calculated action potential and the resting state energy may be helpful for subsequent research or designing the energy consumption of the neuron cluster, such as some research on small-world networks and energy transfer consumption [31–33].

In addition to the energy consumed by the generation of spikes, the propagation of action potentials, and maintaining resting potentials in neurons and glial cells, we ignore other energy consumption by neural networks. Although other energy consumption just accounts for a small part of total energy consumption, our calculations are not accurate enough. It should be noted that the model we used is a simplified coding model. Thus far, how neural networks code information is unknown. Our next work is to refine and calculate the model of neural networks to make it more consistent with the real situation of neuron coding.

## Figures and Tables

**Table 1:** The value of *N* and *A* and the corresponding value of cap and *p*.

All neurons (*N*)	Active neurons (*A*)	cap	cap (normalized)	Ratio (*p*)
100	50	1.0089*e* + 029	1.000	50%
101	45	1.0083*e* + 029	0.999	46%
104	40	9.9480*e* + 028	0.986	38%
116	33	9.9039*e* + 028	0.982	29%
119	32	1.0051*e* + 029	0.996	27%
131	29	9.9655*e* + 028	0.988	22%
143	27	1.0432*e* + 029	1.034	19%
170	24	9.9550*e* + 028	0.987	14%
199	22	1.0015*e* + 029	0.993	11%
219	21	1.0254*e* + 029	1.016	9.5%
276	19	1.0396*e* + 029	1.030	6.9%
373	17	1.0168*e* + 029	1.008	4.6%
558	15	1.0013*e* + 029	0.993	2.7%
971	13	1.0105*e* + 029	1.002	1.3%

## Data Availability

Data is available on request.
